# Association between cardiorespiratory fitness and total brain myelin volume among older adults

**DOI:** 10.1186/s11556-025-00371-0

**Published:** 2025-04-08

**Authors:** Mariusz J. Kujawa, Małgorzata Grzywińska, Angelika K. Sawicka, Anna B. Marcinkowska, Maciej Chroboczek, Zbigniew Jost, Edyta Szurowska, Paweł J. Winklewski, Arkadiusz Szarmach, Sylwester Kujach

**Affiliations:** 1 2nd Department of Radiology, Medical University of Gdansk, Gdansk, 80-214 Poland; 2 Neuroinformatics and Artificial Intelligence Lab, Department of Neurophysiology, Neuropsychology and Neuroinformatics, Medical University of Gdansk, Gdansk, 80-210 Poland; 3 Applied Cognitive Neuroscience Lab, Department of Neurophysiology, Neuropsychology and Neuroinformatics, Medical University of Gdansk, Gdansk, 80-210 Poland; 4 Department of Physiology, Gdansk University of Physical Education and Sport, Gdansk, 80-336 Poland; 5 Department of Biochemistry, Gdansk University of Physical Education and Sport, Gdansk, 80-336 Poland; 6 Department of Neurophysiology, Neuropsychology and Neuroinformatics, Medical University of Gdansk, Gdansk, 80-210 Poland; 7 Institute of Health Sciences, Pomeranian University in Slupsk, Slupsk, 76-200 Poland; 8 Department of Physiology, Medical University of Gdansk, Gdansk, 80-210 Poland

**Keywords:** Myelin, Magnetic resonance imaging, Physical activity, Human brain

## Abstract

**Background:**

Myelin, which insulates neurons, speeds up information transfer and provides the necessary conditions for cognitive and motor functioning. The direct link between physical performance and the total brain myelin volume remains unclear.

**Methods:**

This study involved 87 healthy participants (71 women, 16 men) with a mean age of 69.3 ± 3.14 years and a mean body mass index of 27.83 ± 3.93 kg/m^2^. Several measures of physical fitness (isometric muscle strength, handgrip strength, and cardiopulmonary exercise testing) were examined for their correlations with the total brain myelin volume using Synthetic MRI, an FDA-approved myelin assessment software.

**Results:**

A high maximal respiratory exchange ratio and low maximal heart rate achieved during cardiopulmonary exercise testing were associated with higher estimated brain myelin content. In addition, the handgrip strength test performance as well as the peak and average peak torque were associated with higher brain parenchymal myelin volumes.

**Conclusions:**

We demonstrated that higher brain myelin content was positively associated with better cardiorespiratory fitness and higher upper and lower limb muscle strength in older individuals. These findings provide new insights into the development of improved rehabilitation and exercise schemes to preserve cognitive health in the older adult population.

**Supplementary Information:**

The online version contains supplementary material available at 10.1186/s11556-025-00371-0.

## Background

Lipid-rich myelin sheaths insulate nerve cell axons, providing protection and ensuring that action potentials are conducted without decrement. Myelin is thus indispensable for proper information transfer that provides the necessary conditions for cognitive binding and motor actions [1–3]. The myelin volume increases during brain development. The highest brain myelin content (MYC) is reached between 30 and 60 years of age, after which it gradually declines [4–7]. Demyelination of the brain is recognised as a hallmark of Alzheimer’s disease [8]. The total brain myelin volume can thus serve as a valuable tool to assess the progression of neurodegenerative processes associated with physiological ageing [9].

Synthetic MRI (SyMRI) (SyntheticMR, Linköping, Sweden) is a CE-marked and FDA-cleared magnetic resonance imaging (MRI) software for clinical applications. It uses a single 5- to 6-minute sequence to acquire information regarding the brain tissue properties and further calculate the volumes of the whole brain, white matter, grey matter, cerebrospinal fluid, and myelin as well as the brain parenchymal fraction [10–12]. The SyMRI automatic myelin detection system was validated in 12 histological samples of human cadaver brains, and a correlation of *r* = 0.74 between the histological myelin marker and the SyMRI myelin maps was reported [13].

Physical activity can counteract a number of abnormalities associated with brain ageing that lead to myelin loss in humans [8]such as reduced availability of trophic factors [14–16], reduced cerebral perfusion [17], loneliness and health anxiety leading to depression [18, 19], and a general decline in electrical activity in the brain due to reduced activities of daily living [20–22]. Animal studies show that aerobic exercise can restore the production of cholesterol [23], a key component of myelin, and promote the differentiation and maturation of oligodendrocytes [24]. These mechanisms operate alongside neuro-supporting tissues, including glial tissue and oligodendrocytes involved in the myelination process [25]. Convincing evidence suggests that physical activity and, more broadly, an active lifestyle are associated with an improved brain myelin profile in humans [26]. Nevertheless, the association between physical performance and the brain myelin volume remains unclear [26].

Previous studies have also shown an association between cardiorespiratory fitness and cognitive function among older adults [33]. Individuals who maintain higher levels of cardiorespiratory fitness tend to exhibit better cognitive performance than their less fit counterparts [34]. Interestingly, it was the cardiovascular fitness and sleep quality, but not amount of physical activity itself, total sleep time or lower body fat, that were associated with increased functional connectivity within key resting state networks [27]. Because of the direct link between age-related changes in myelination and information processing speed, brain myelin content is increasingly recognised as a sensitive biomarker for interventions targeting age-related neurodegenerative changes [28].

Therefore, we aimed to identify the physical performance markers associated with the total brain myelin volume. We hypothesised that cardiorespiratory fitness parameters would be associated with the total MYC in older adults. As there are no studies on myelin markers and their associations with strength/resistance markers of physical fitness, we conducted an exploratory study.

## Methods

### Participants

Older adults were recruited from Gdansk and nearby regions of northern Poland through advertisements in senior citizen clubs, clinics, third-age universities, and social media. One hundred eighty people applied for enrolment. After an initial interview and screening tests, 87 participants (71 women and 16 men) were included in the present study (mean age, 69.3 ± 3.14 years; mean body mass index [BMI], 27.83 ± 3.93 kg/m^2^). The criteria for inclusion in the study were an age of 62–75 years and low to moderate daily physical activity. Individuals with any contraindication to magnetic resonance imaging (MRI) or the presence of chronic diseases (such as cancer, severe heart disease, history of myocardial infarction or stroke, musculoskeletal system diseases that prevent daily activity, diabetes, neurological and psychiatric diseases, or other conditions requiring psychopharmacological or anti-inflammatory drug treatment) were excluded.

This study was approved by the Medical University of Gdansk Bioethical Committee (NKBBN/499/2021). All participants were informed about the study protocol and provided written informed consent before the assessment.

### Anthropometric measurements

Body mass and body composition were assessed using a multi-frequency impedance body composition analyser (InBody 720; InBody Co., Ltd., Seoul, Korea). This device precisely evaluates body water and body composition, providing detailed measurements of fat mass, free fat mass, skeletal muscle mass, and soft lean mass [29].

### Muscle strength assessment

Isometric muscle strength was determined using a Biodex System 4 dynamometer (Biodex Medical Systems, Inc., Shirley, NY, USA). Peak torque was measured during a 5-second isometric contraction with both flexion and extension at the knee joint. After receiving a detailed explanation of the assessment, each participant completed one set of submaximal contractions to become comfortable with the testing procedure. After a 10-minute warm-up, each participant was positioned on the apparatus in accordance with the manufacturer’s manual. Each test was performed while the participant was seated, with straps stabilising their lower limbs and trunk. The knee and hip joints were positioned at a 90-degree angle to obtain knee torque. The participant received verbal encouragement to reach their full potential during each measurement. Using a Compaq Deskpro laptop (Compaq, Palo Alto, CA, USA) and the Biodex programme, data were gathered in accordance with the Biodex standard protocol [16].

### Handgrip strength

Handgrip strength was assessed using a handheld calibrated digital hand dynamometer (SAEHAN DHD-1; SAEHAN Corporation, Changwon, South Korea). The handle width was adjusted to the participant’s hand size. The participants stood with their arms parallel to their trunk and were encouraged to squeeze the dynamometer as hard as possible. The variables of maximal hand grip strength and average strength were used for analysis. Each participant performed three attempts per side with a rest period before each attempt. Each attempt was assessed in either the right or left hand. The results were not stratified by dominant hand because maximal values are not always reached in the dominant hand [30]. The reliability of dynamometer-based strength measurement has been presented elsewhere [31].

### Cardiopulmonary exercise testing

All participants underwent cardiopulmonary exercise testing in the upright position on an electronically braked cycle ergometer (ViaSprint 150P; ergoline GmbH, Bitz, Germany). Following a 5-minute warm-up at an intensity of 30 W for women and 50 W for men, participants began the peak oxygen uptake (VO₂peak) testing. The exercise intensity was increased incrementally by 15 W/min until exhaustion (test phase). Participants were required to maintain a pedalling frequency of 55 to 65 revolutions per minute (rpm) throughout the test. Pulmonary gas exchange was monitored breath-by-breath using Jaeger Oxycon Champion equipment (Viasys Healthcare, Höchberg, Germany), with measurements averaged at 10-second intervals. VO₂peak was determined as the highest 30-second rolling mean prior to test termination [32]. The maximal respiratory exchange ratio (RER_max_) was also recorded during testing. The values of VO_2_ and VCO_2_ were used to calculate the RER, which is the ratio of exhaled CO_2_ to inhaled O_2_ and is an indirect measure of the oxidative capacity of skeletal muscle to produce energy [33]. Heart rate (HR, b·min⁻¹) was continuously monitored using telemetry (Polar Electro-Oy, Finland). The maximal load achieved in the last fully completed stage was recorded as the maximal aerobic power (MAP), serving as the aerobic capacity parameter in this study.

Before each test, the gas analyzer was calibrated using standard gases of known concentrations, following the manufacturer’s guidelines.

### Myelin compounds

The scans were conducted using a 3T Magnetom Vida MRI system (Siemens Healthineers, Erlangen, Germany) equipped with a head/neck 20-channel coil. Myelin mapping was performed with SyMRI software, which employs a specialized scanning sequence to precisely measure various brain properties, including T1-weighted, T2-weighted, and inversion times. The SyMRI scanning process utilizes a fast, multi-echo, multi-delay acquisition method. The sequence type is a fast spin-echo or gradient echo, customized explicitly for SyMRI. Acquisition parameters include a repetition time (TR) of 4–5 s and an echo time (TE) ranging from 20 ms to 120 ms. The voxel size is 1.2 × 1.2 × 4 mm, with a slice thickness of 4 mm and a matrix size 256 × 256. The total scan time for the procedure is approximately 5 min.

The SyMRI software performs automated tissue segmentation and volumetric assessments. Each voxel is categorized into contrast compartments for myelin, cellular, and water partial volumes based on distinct relaxation properties. The software quantifies the myelin-correlated volume (MYC) and other brain tissue properties. SyMRI implements a unique scanning sequence that precisely measures various brain properties. This technology enables the creation of 12 adjustable contrast-weighted images by modifying the echo time, repetition time, and inversion time. In addition, SyMRI performs automated tissue segmentation and volumetric assessments.

Within the SyMRI framework, each voxel is categorised into four contrast compartments according to the myelin model: myelin partial volume (VMY), cellular partial volume (VCL), free water partial volume, and excess parenchymal water partial volume. The allocation for each compartment ranges from 0 to 100%, with their sum always reaching 100%. These compartments are characterised by unique relaxation attributes delineated by a specific longitudinal relaxation rate, transverse relaxation rate, and proton density. The quantitative acquisition sequence of SyMRI captures these values and incorporates them into the model. The VMY compartment encompasses the myelin water and sheaths tightly surrounding axons, leading to rapid relaxation because of their proximity.

By contrast, the VCL compartment includes a mixture of intracellular and extracellular water, axonal water, and cellular macromolecules unrelated to myelin, resulting in a median relaxation time of VCL that is slower than that of VMY but faster than that of the free water partial volume. Magnetisation exchange is observed between VMY and VCL. Normally, brain parenchyma voxels contain a combination of VMY and VCL, with grey and white matter voxels exhibiting lower and higher myelin partial volumes, respectively [9, 13].

Quality assurance (QA) metrics for segmentation involve software-specific evaluations to ensure accuracy and reliability. One commonly used metric is the Dice Similarity Coefficient (DSC), which compares SyMRI segmentation outputs to manually labeled ground truth or another validated segmentation method; DSC values of 0.85 or higher. Another critical metric is the Hausdorff Distance (HD), which evaluates boundary agreement by measuring the maximum distance between the edges of the segmentation and the ground truth.

Visual inspection of segmentation involves two key aspects: overlay assessment and anatomical accuracy. For overlay assessment, segmentation results are overlaid onto the corresponding synthetic images, such as T1-weighted or T2-weighted images, to evaluate their alignment. It is essential to ensure that segmentation boundaries closely follow anatomical structures. Regarding anatomical accuracy, the segmentation should respect major anatomical landmarks. This includes ensuring that gray matter boundaries align with cortical folds, ventricles are delineated as CSF and subcortical structures, if segmented, correspond to their known shapes.

### Statistical analysis

All analyses were conducted using SPSS software (version 26.0, IBM Corp.). A two-tailed p-value of ≤ 0.05 was considered statistically significant unless adjustments for multiple comparisons were applied. Descriptive statistics summarized participants’ characteristics, with continuous variables presented as mean ± standard deviation (SD) and categorical variables as percentages.

The dataset’s distribution was evaluated using normality tests, including the Kolmogorov-Smirnov test, to assess whether the data followed a normal distribution. Non-parametric tests were employed for variables that did not meet normality assumptions. Correlation analyses were performed using the Spearman correlation method to examine relationships between physiological measurements and myelin-related variables.

Group comparisons were conducted using one-way ANOVA among multiple groups. These analyses focused on distinctions in BMI, VO₂peak, and myelin levels. Regression analyses were performed to explore the relationships between myelin levels and various independent variables, with coefficients and R² values reported to quantify the proportion of variance explained by the predictors.

Additionally, cross-sectional and comparative analyses examined group-level trends, such as the association between BMI quartiles and mean myelin levels.

### PostHoc power analysis

A post-hoc power analysis was conducted using G*Power [34]. The analysis assumed a medium effect size (*r* = 0.30) and a significance level of α = 0.05. The total sample size was 70. The achieved power was 0.83, indicating that the test had an 83% chance of detecting an effect of this size if such an effect exists. The obtained power exceeded the commonly recommended threshold of 0.80 [35], suggesting that the sample size was sufficient to conduct a reliable statistical analysis.

## Results

### Participants’ characteristics

Descriptive statistics of the study population are provided in Table 1.

**Table 1:** Descriptive statistics of the study population and results

VARIABLES		Mean	SD
**Anthropometric**			
Age		69.30	3.14
Height [cm]		161.57	7.03
Weight [kg]		72.75	11.70
BMI [kg·m^− 2^]		27.83	3.93
**Aerobic capacity**			
VO2peak [mL·min^− 1^·kg^− 1^]		22.93	5.18
MAP [W]		118.43	28.57
HRmax [beat ·min^− 1^]		146.71	17.58
RERmax		1.20	0.11
**Anaerobic capacity**			
Peak Torque -away [Nm]		127.93	40.35
Peak Torque -towards [Nm]		48.96	16.03
Average Peak Torque -away [Nm]		121.13	38.75
Average Peak Torque -towards [Nm]		46.07	16.14
Hand grip strength – Left [kG]		28.18	7.80
Hand grip strength – Right [kG]		30.66	8.53
**Myelin compound**			
Myelin fraction (MYC/ICV)		13.07	0.96
Myelin fraction (MYC/BPV)		10.71	0.98
Myelin-correlated compound [ml]		149.16	17.21
Brain parenchymal volume [ml]		1140.00	84.67
Intracranial volume [ml]		1393.56	106.09
Brain parenchymal volume [%]		13.06	0.94
Intracranial volume [%]		10.71	0.97
Brain parenchymal fraction (BPV/ICV) [%]		81.88	3.19

SD – standard deviation, BMI – body mass index, VO2peak – peak oxygen uptake, MAP – maximal aerobic power, HRmax – maximal heart rate, RERmax – maximal respiratory exchange ratio

The proportions of myelin relative to intracranial volume and brain parenchymal volume (MYC/ICV and MYC/BPV) are close in range. The low standard deviations in both cases suggest that the participants show relatively consistent levels of myelin fraction, regardless of whether it is referenced to ICV or BPV (Table 1).

### Correlation analysis between Myelin compound and anthropometric variables

Positive correlations were identified between MYC and height as well as between MYC and brain parenchymal volume. The intracranial volume was positively associated with age, height, weight, and BMI. The brain parenchymal fraction was negatively correlated with age. These results suggest that brain volume measures (both parenchymal and intracranial) are consistently associated with anthropometric characteristics such as height, weight, water mass, and lean mass, whereas myelin-related measures showed weaker or nonsignificant correlations. The positive correlations with body composition parameters highlight a potential link between brain structure and overall body composition (Table 2).

**Table 2:** Results of correlation analysis between Myelin compound and anthropometric variables

*R* ^2^	Age	Height [cm]	Weight [kg]	Intracellular Water Mass	Extracellular Water Mass	Protein Mass	Mineral Mass	Body Fat Mass	Total Body Water Mass	Skeletal Lean Mass	Fat Free Mass
Myelin-correlated compound [ml]	0.05	**0.26** ^*^	0.09	0.21	0.21	0.21	0.22	-0.06	0.21	0.20	0.21
Myelin fraction (MYC/BPV)	-0.09	0.01	-1.14	-0.05	-0.05	-0.05	-0.01	-0.16	-0.05	-0.05	-0.05
Myelin fraction (MYC/ICV)	-0.16	-0.04	-0.10	-0.08	-0.09	-0.08	-0.03	-0.04	-0.09	-0.08	-0.08
Brain parenchymal volume [ml]	0.15	**0.38** ^**^	0.259^*^	**0.35** ^**^	**0.36** ^**^	**0.36** ^**^	**0.34** ^**^	0.04	**0.36** ^**^	**0.35** ^**^	**0.35** ^**^
Intracranial volume [ml]	**0.27** ^*^	**0.40** ^**^	**0.257** ^*^	**0.41** ^**^	**0.41** ^**^	**0.41** ^**^	**0.37** ^**^	-0.04	**0.41** ^**^	**0.40** ^**^	**0.40** ^**^
Brain parenchymal volume [%]	-0.09	0.01	-0.14	-0.04	-0.04	-0.04	-0.01	-0.16	-0.05	-0.04	-0.04
Intracranial volume [%]	-0.16	-0.04	-0.09	-0.08	-0.08	-0.08	-0.02	-0.03	-0.08	-0.08	-0.08
Brain parenchymal fraction (BPV/ICV) [%]	**-0.26**	-0.13	-0.02	-0.15	-0.17	-0.15	-0.10	0.15	-0.15	-0.15	-0.15

R^2^-correlation coefficient, * *p* ≤ 0.05, ** *p* ≤ 0.01

### Correlation analysis between Myelin compound and aerobic capacity

Positive correlations were identified between MAP and MYC, brain parenchymal volume, and intracranial volume. The maximal respiratory exchange ratio (RER_max_) was positively associated with MYC, brain parenchymal volume, and intracranial volume. Both MYC/ICV and MYC/BPV show a consistent moderate positive correlation with the respiratory exchange ratio (RER_max_) and a negative correlation with maximal heart rate (HR_max_). However, minimal correlations were observed between myelin fractions and measures of aerobic capacity, such as VO_2peak_ and maximal aerobic power (MAP), indicating that aerobic fitness levels have limited association with myelin fraction in this dataset (Table 3).

**Table 3:** Results of correlation analysis between Myelin compound and aerobic capacity parameters

*R* ^2^	VO2peak	MAP	HRmax	RERmax
Myelin-correlated compound [ml]	0.18	0.24^*^	0.04	0.25^*^
Myelin fraction (MYC/BPV)	0.10	-0.15	-0.26*	0.33*
Myelin fraction (MYC/ICV)	0.05	-0.22	-0.29*	0.32*
Brain parenchymal volume [ml]	0.12	0.24^*^	-0.02	0.07
Intracranial volume [ml]	0.13	0.283^*^	-0.02	-0.04
Brain parenchymal volume [%]	0.10	0.09	0.05	**0.32** ^**^
Intracranial volume [%]	0.05	0.02	0.05	**0.32** ^**^
Brain parenchymal fraction (BPV/ICV) [%]	-0.07	-0.16	0.00	0.16

R^2^-correlation coefficient, VO2peak – peak oxygen uptake, MAP – maximal aerobic power, HRmax – maximal heart rate, RERmax – maximal respiratory exchange ratio, * *p* ≤ 0.05, ** *p* ≤ 0.01

**Fig. 1:**
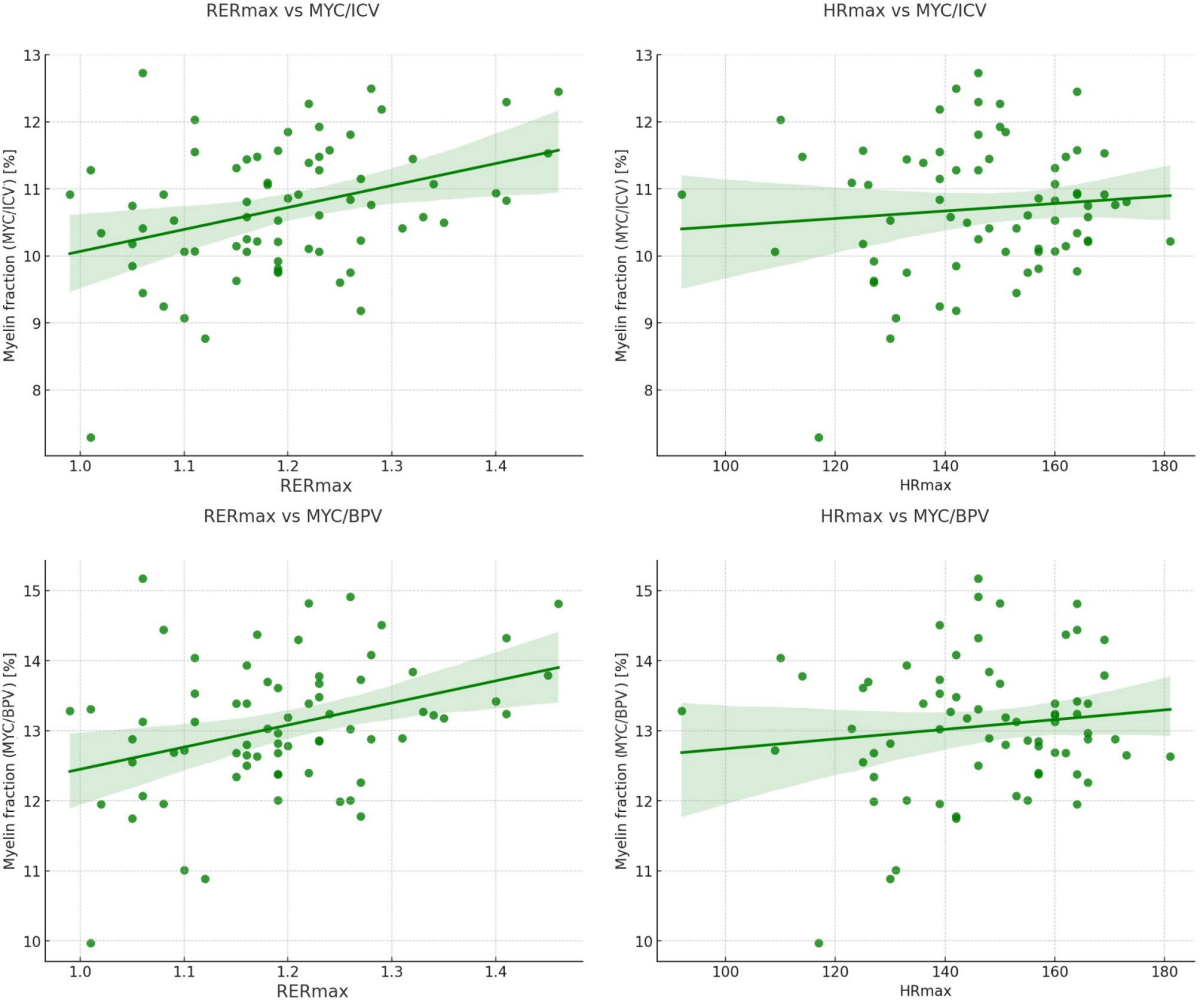
Association between myelin compound and cardiorespiratory fitness. (**a**) RER_max_ vs. MYC/ICV, (**b**) RER_max_ vs. MYC/BPV (**c**) HR_max_ vs. MYC/ICV, (**d**) HR_max_ vs. MYC/BVP. Both, the proportions of myelin relative to intracranial volume and brain parenchymal volume MyC/ICV and MyC/BPV show a consistent moderate positive correlation with the respiratory exchange ratio (RER_max_) and a negative correlation with maximal heart rate (HR_max_)

### Correlation analysis between Myelin compound and anaerobic capacity

A positive correlation was identified between MYC and handgrip strength. Positive correlations were also shown between brain parenchymal volume and peak torque-away, average peak torque, and handgrip strength as well as between intracranial volume and peak torque-away, average peak torque, and handgrip strength. Myelin Fraction (MYC/BPV) shows a moderate positive trend with peak torque away from the body (R² = 0.43). However, it weakly correlates with hand grip strength and other torque measures. In contrast, myelin fraction (MYC/ICV) displays no meaningful correlations with physical performance measures (Table 4).

**Table 4:** Results of correlation analysis between Myelin compound and anaerobic capacity parameters

R^2^	Peak Torque -away [Nm]	Peak	Average Peak Torque -away [Nm]	Average	Hand grip strength	Hand grip strength
		Torque		Peak Torque	-Left [kG]	-Right [kG]
		-towards [Nm]		-towards [Nm]		
Myelin-correlated compound [ml]	0.23	0.04	0.22	0.06	0.16	0.28^*^
Myelin fraction (MYC/BPV)	0.43	-0.16	0.03	-0.14	0.04	0.07
Myelin fraction (MYC/ICV)	-0.01	-0.11	-0.2	-0.09	0.02	0.04
Brain parenchymal volume [ml]	0.27^*^	0.16	0.2^*^	0.17	0.20	0.35^**^
Intracranial volume [ml]	**0.31** ^**^	0.15	**0.30** ^*^	0.17	0.20	**0.35** ^**^
Brain parenchymal volume [%]	0.04	-0.16	0.03	-0.13	0.04	0.07
Intracranial volume [%]	0.00	-0.11	-0.01	-0.09	0.01	0.04
Brain parenchymal fraction (BPV/ICV) [%]	-0.16	-0.04	-0.16	-0.04	-0.04	-0.06

R^2^-correlation coefficient, * *p* ≤ 0.05, ** *p* ≤ 0.01

Most of the participants were slightly overweight. MYC had a moderate positive correlation with height (0.229) and a weaker correlation with weight and BMI. Weight shows a strong negative association with MYC/BPV.

There were no differences in MYC across BMI percentiles in the investigated population (Table 5). The mean MYC was 151.19 mL and 148.97 mL for Q1 and Q3, respectively.

**Table 5:** Mean and standard deviation for two BMI quartiles (Q1 and Q3) in relation to MYC levels

VARIABLES		Mean	SD
**Myelin-correlated compound (MyC) [ml**			
Q1 BMI		151.19	13.86
Q3 BMI		148.97	19.22

SD-standard deviation, BMI-body mass index

## Discussion

The two main findings of this study are as follows. First, the parameters of cardiorespiratory fitness (high RER_max_, low HR_max_) were associated with a higher myelin fractions (MYC/ICV and MYC/BPV). Second, the peak torque was associated with higher myelin fraction (MYC/BPV).

The ageing process is naturally associated with the loss not only of physical fitness, as evidenced by declines in aerobic capacity and muscle strength correlated with sarcopenia, but also with a decline in cognitive performance [36, 37]. Animal studies and human experiments using advanced neuroimaging tools indicate that the decline in cognitive performance is accompanied by structural changes in the brain [25, 37–39]. These findings were largely confirmed in our study as indicated by a decrease in the brain parenchymal fraction with age.

Intriguingly, a similar decrease in skeletal muscle strength has been shown to coincide with changes in the structure of skeletal muscle (loss of skeletal muscle mass together with an increase in adipose tissue) [40]. Therefore, both pharmacological and non-pharmacological therapies are still being sought to influence the structural and consequently functional changes in the brains of older adults.

Myelin, a complex blend of proteins and lipids, with lipids comprising approximately 70–85% of its dry weight, is indirectly detected by MRI through T2 relaxation analysis. The presence of a short T2 component suggests that water is trapped within myelin layers. MYC is a product of a novel model that estimates myelin’s partial volume by examining its effect on water inside and outside cells with the use of rapid quantitative MRI. This method indirectly assesses myelin by measuring its impact on specific MRI parameters [13, 41, 42]. In our study, the MYC was then scaled to total intracranial volume and brain parenchymal volume (MYC/ICV and MYC/BPV) to adjust for individual differences in head size intracranial volume.

In a cross-over study, Boa Sorte Silva et al. [43] demonstrated that physical activity in older adults with mild cerebrovascular pathology was positively associated with greater MYC in the brain white matter. The assessment of the participants’ physical activity was based on an established questionnaire titled ‘Physical Activity Scale for the Elderly’. The results of other longitudinal studies have indicated that high-intensity aerobic exercise (such as cycling, walking, or dancing) augments myelination in brain regions related to a particular exercise scheme (i.e., motor cortex) or late myelinating regions that are at highest risk of ageing-related demyelination [26, 44–46]. All the above-mentioned studies except that by Rowley et al. [44] assessed the myelin fraction in brain white matter.

The present study demonstrates that better cardiorespiratory fitness performance (as indicated by RER_max_ and HR_max_ values) is associated with an improved whole-brain myelination index (measured with MYC/ICV and MYC/BPV). Subsequent studies have linked white matter microstructural integrity to the rate of cognitive decline in normative ageing. Thus, MRI myelin metrics, which are relatively easy to assess, appear not only to reflect microstructural changes but also to be potentially predictive of cognitive trajectories [47, 48]. As a result, MRI metrics could become a useful tool to be used in cardiovascular rehabilitation programmes [49].

In particular we showed a significant positive correlation between the RER_max_ achieved during the cardiopulmonary test and the MYC/ICV and MYC/BPV. To the best of our knowledge we are the first to demonstrate such association. We speculate that an increased RER_max_ may indicate increased buffering efficiency. Because of the sensitivity of tissues to low pH, more efficient removal of H^+^ ions could have a protective effect on both neurons and MYC. Although RER does not directly correlate with VO_2max_, its association with various indicators of physical fitness has been established [33, 50].

Maximum (or peak) heart rate (HR_max_), in turn, refers to the upper limit of what the cardiovascular system can handle during physical activity or a cardiopulmonary exercise test. It can therefore be considered an easily measured indicator of physical fitness [51].

Upper limb muscle strength has been shown to be associated with better functioning in selected cognitive domains (e.g., verbal learning and memory) in nursing home residents [52]. The handgrip test, historically used to detect autonomic neuropathy of diabetic origin, has recently been associated with parameters of hypertension-related target organ damage. Because of its simplicity, the handgrip might be useful as a screening tool to identify patients with high cardiovascular risk [53]. The relationship between cardiovascular health and cognitive functioning in adults, although very intuitive [17], has been supported by very few experimental studies to date [39, 54]. In our study MYC/ICV and MYC/BPV were weakly correlated with hand grip strength.

Another study showed that 12 weeks of lower limb muscle strength training was positively correlated with performance of the digit symbol substitution test [55]. However, more general neuropsychological tests do not seem able to detect cognitive changes evoked by lower limb muscle strength [56]. A cross-sectional study involving functional near-infrared spectroscopy showed no relationship between lower limb strength and either the level of oxygenated haemoglobin in the prefrontal cortex or improvement of cognition [57]. Therefore, the relationship between lower limb muscle strength and cognitive functioning requires further investigation. We demonstrated for the first time that lower limb strength is positively correlated with the total myelin brain parenchymal and intracranial volumes fractions.

Interestingly, body weight shows a strong negative association with MYC/BPV, possibly indicating that body composition, rather than absolute body weight, plays a key role in explaining variations in myelin fractions. However, we did not find an association with body fat mass or skeletal lean mass.

Most of the participants were slightly overweight. This particular study group composition might be seen as a limitation. However, it represents a very typical pattern of Western societies. Another potential weakness is the imbalance between male and female participants. As less than 20% of participants were male, this may limit the generalisability of the findings. The main strengths of our study are the large investigated population, performance of physical fitness testing that included several measures, and myelin assessment with FDA-approved technology.

In conclusion, we have shown that better cardiorespiratory fitness and higher limb muscle strength are associated with higher brain MYC in older adults. These findings may pave the way for the development of more effective rehabilitation strategies that preserve cognitive health in older adults. MRI myelin-based metrics may offer new ways to assess the effectiveness of such strategies.

## Electronic supplementary material

Below is the link to the electronic supplementary material.

Supplementary Material 1

## Data Availability

The datasets used and/or analysed during the current study are available from the corresponding author on reasonable request.
