# Supplementary material for: Association between cardiorespiratory fitness and total brain myelin volume among older adults
Source: Eur Rev Aging Phys Act. 2025 Apr 8;22:5. doi: 10.1186/s11556-025-00371-0 (PMC11980140; doi:10.1186/s11556-025-00371-0)
Supplement: Supplementary file 1 — Supplementary Material 1 [file 11556_2025_371_MOESM1_ESM.docx]

Reviewer #1: The authors have adequately addressed all my comments. Therefore, I consider the manuscript acceptable for publication.

**Thank you for your kind words!**

Reviewer #2: This is a very interesting study with high clinical and practical significance.

My comments are listed below:

PostHoc Power Analysis: The authors should perform a post-hoc power analysis in order to declare the power of their sample.

**PostHoc Power Analysis: The authors should perform a post-hoc power analysis in order to declare the power of their sample.**

A post-hoc power analysis was conducted using G*Power (Faul, Erdfelder, Lang, & Buchner, 2007). The analysis assumed a medium effect size (r = 0.30) and a significance level of α = 0.05. The total sample size was 70. The achieved power was 0.83, indicating that the test had an 83% chance of detecting an effect of this size if such an effect exists. The obtained power exceeded the commonly recommended threshold of 0.80 (Cohen, 1988), suggesting that the sample size was sufficient to conduct a reliable statistical analysis.

There is no need for Table 2 where the results of correlation analysis between myelin compound and anthropometric variables are presented. Instead, the authors should consider presenting the data from body composition measurements such as %Lean Body Mass, or Fat mass or TBM etc. Those indices are more interesting compared to height and weight.

**The requested variables have been added to Table 2.**

**These results suggest that brain volume measures (both parenchymal and intracranial) are consistently associated with anthropometric characteristics such as height, weight, water mass, and lean mass, whereas myelin-related measures showed weaker or nonsignificant correlations. The positive correlations with body composition parameters highlight a potential link between brain structure and overall body composition.**

**Table 2.** Results of correlation analysis between myelin compound and anthropometric variables

| R^2^ | | Age | Height [cm] | Weight [kg] | Intracellular Water Mass | Extracellular Water Mass | Protein Mass | Mineral Mass | Body Fat Mass | Total Body Water Mass | Skeletal Lean Mass | Fat Free Mass |
| --- | --- | --- | --- | --- | --- | --- | --- | --- | --- | --- | --- | --- |
|  | Myelin-correlated compound [ml] | 0.05 | **0.26^*^** | 0.09 | 0.21 | 0.21 | 0.21 | 0.22 | -0.06 | 0.21 | 0.20 | 0.21 |
|  | **Myelin fraction (MYC/BPV)** | -0.09 | **0.01** | -1.14 | -0.05 | -0.05 | -0.05 | -0.01 | -0.16 | -0.05 | -0.05 | -0.05 |
|  | **Myelin fraction (MYC/ICV)** | -0.16 | **-0.04** | -0.10 | -0.08 | -0.09 | -0.08 | -0.03 | -0.04 | -0.09 | -0.08 | -0.08 |
|  | Brain parenchymal volume [ml] | 0.15 | **0.38^**^** | 0.259^*^ | **0.35^**^** | **0.36^**^** | **0.36^**^** | **0.34^**^** | 0.04 | **0.36^**^** | **0.35^**^** | **0.35^**^** |
|  | Intracranial volume [ml] | **0.27^*^** | **0.40^**^** | **.257^*^** | **0.41^**^** | **0.41^**^** | **0.41^**^** | **0.37^**^** | -0.04 | **0.41^**^** | **0.40^**^** | **0.40^**^** |
|  | Brain parenchymal volume [%] | -0.09 | 0.01 | -0.14 | -0.04 | -0.04 | -0.04 | -0.01 | -0.16 | -0.05 | -0.04 | -0.04 |
|  | Intracranial volume [%] | -0.16 | -0.04 | -0.09 | -0.08 | -0.08 | -0.08 | -0.02 | -0.03 | -0.08 | -0.08 | -0.08 |
|  | Brain parenchymal fraction (BPV/ICV) [%] | **-0.26** | -0.13 | -0.02 | -0.15 | -0.17 | -0.15 | -0.10 | 0.15 | -0.15 | -0.15 | -0.15 |
|  | R^2^-correlation coefficient, * *p ≤ 0.05,* ** *p ≤ 0.01* | | | |  |  |  |  |  |  |  |  |
